# CASUAL EFFECTS OF EARLY-LIFE ADVERSITY AND MID/LATE-LIFE BEHAVIORAL INTERVENTION ON THE PACE OF BIOLOGICAL AGING

**DOI:** 10.1093/geroni/igad104.0676

**Published:** 2023-12-21

**Authors:** Daniel Belsky, Luigi Ferrucci

## Abstract

Epigenetic clocks have taken gerontology by storm. With the advent of 2nd and 3rd generation epigenetic clocks, including GrimAge and DunedinPACE, the field now has genomic biomarkers of aging that exhibit strong and consistent associations with aging-related morbidity and mortality. But the causes of variation in these powerful indices of aging-related biological changes remain poorly understood. In this symposium, we present four studies at the vanguard of research to elucidate causal drivers of 2nd and 3rd generation epigenetic clocks drawn from four disciplines. From life-course epidemiology, we present a natural-experiment study of the long-term impact of in-utero famine exposure. From economics, we present a quasi-natural experiment study of long-term impacts of early-life exposure to the Great Depression. From psychology, we present results from a randomized controlled trial (RCT) of cognitive behavioral therapy (CBT) for insomnia in older adults. And from geroscience, we present results from an RCT of long-term calorie restriction in healthy, non-obese adults (CALERIE). These studies report novel and important findings that suggest early-life adversity acts to accelerate the pace of biological aging –and— that it may be possible to slow the pace of biological aging in mid- and later-life through behavioral intervention. They also illustrate study designs and methods that can help move the field forward as costs for measurement of epigenetic clocks fall, enabling their introduction into many more cohort and intervention studies.

